# Tead1 is required for perinatal cardiomyocyte proliferation

**DOI:** 10.1371/journal.pone.0212017

**Published:** 2019-02-27

**Authors:** Ruya Liu, Rajaganapathi Jagannathan, Feng Li, Jeongkyung Lee, Nikhil Balasubramanyam, Byung S. Kim, Ping Yang, Vijay K. Yechoor, Mousumi Moulik

**Affiliations:** 1 Division of Diabetes, Endocrinology and Metabolism, Department of Medicine, University of Pittsburgh, Pittsburgh, Pennsylvania, United States of America; 2 Division of Diabetes, Endocrinology and Metabolism, Department of Medicine, Baylor College of Medicine, Houston, Texas, United States of America; 3 Division of Cardiology, Department of Pediatrics, University of Pittsburgh, Pittsburgh, Pennsylvania, United States of America; 4 Division of Cardiology, Department of Pediatrics, UTHealth McGovern Medical School, Houston, Texas, United States of America; Murdoch Childrens Research Institute, AUSTRALIA

## Abstract

Adult heart size is determined predominantly by the cardiomyocyte number and size. The cardiomyocyte number is determined primarily in the embryonic and perinatal period, as adult cardiomyocyte proliferation is restricted in comparison to that seen during the perinatal period. Recent evidence has implicated the mammalian Hippo kinase pathway as being critical in cardiomyocyte proliferation. Though the transcription factor, Tead1, is the canonical downstream transcriptional factor of the hippo kinase pathway in cardiomyocytes, the specific role of Tead1 in cardiomyocyte proliferation in the perinatal period has not been determined. Here, we report the generation of a cardiomyocyte specific perinatal deletion of Tead1, using Myh6-Cre deletor mice (Tead1-cKO). Perinatal Tead1 deletion was lethal by postnatal day 9 in Tead1-cKO mice due to dilated cardiomyopathy. Tead1-deficient cardiomyocytes have significantly decreased proliferation during the immediate postnatal period, when proliferation rate is normally high. Deletion of Tead1 in HL-1 cardiac cell line confirmed that cell-autonomous Tead1 function is required for normal cardiomyocyte proliferation. This was secondary to significant decrease in levels of many proteins, in vivo, that normally promote cell cycle in cardiomyocytes. Taken together this demonstrates the non-redundant critical requirement for Tead1 in regulating cell cycle proteins and proliferation in cardiomyocytes in the perinatal heart.

## Introduction

Mammalian adult heart size is achieved by a combination of proliferation (hyperplasia) and an increase in cardiomyocyte size (hypertrophy). Cardiomyocytes proliferate at a high rate in the perinatal period, setting a range for the eventual cardiomyocyte cell number in the adult heart. Shortly after birth, proliferation declines drastically, physiological hypertrophy, in turn, constitutes the major mechanism of further heart growth [1]. Similar to other highly specialized post mitotic cells, cardiomyocyte proliferation is restricted in the adult heart, thus limiting regeneration after injury. Therefore, gaining more insights into and understanding the molecular mechanisms underlying cardiomyocyte proliferation is critical towards developing cardiomyocyte replacement as potential therapeutic approach for cardiac diseases.

The highly conserved Hippo-Tead signaling pathway, which regulates cell proliferation and apoptosis, has emerged as one of the very important regulators of organ size control [2]. The inhibitory Hippo signaling pathway is activated by high cell density and other extracellular cues to Mst kinases 1/2 (mammalian STE20-like protein kinase)-Sav1 (Salvador homolog 1) complex, which gets activated and subsequently phosphorylates and activates Lats kinases 1/2 (large tumor suppressor kinase)-Mob1α/1β (Mob kinase activator 1). Lats1/2, in turn, phosphorylates transcriptional co-activators Yap and Taz, which are then sequestered in the cytoplasm via association with 14-3-3 family members, and then degraded in a proteasome-dependent manner. In the absence of this inhibitory phosphorylation by the Hippo kinase pathway, Yap/Taz translocate to the nucleus and bind to transcription factors, including Tead1, to induce genes promoting cell cycle and survival. Inactivation of Hippo pathway–either by silencing upstream kinases such as Mst1/2, Lats2 or its binding partner Salvador [3], or by activation of downstream kinase effectors Yap [1, 4]–resulted in an increased heart size and cardiomyocyte number at both embryonic and postnatal stages with evidence of regenerative myocardium post-injury [5, 6]. While all these studies demonstrate the importance of the mammalian hippo kinase components including Yap, the downstream transcriptional effector has not been conclusively demonstrated. Mechanistically, Yap protein, as a co-activator, possesses a transcriptional activation domain, but lacks a DNA binding domain. Hence, it requires other DNA-binding transcription factors to regulate transcription, of which the Tead family serve as the major transcriptional effectors [7], with one study demonstrating that Tead1-Yap interaction was required for the proliferative effects of Yap1 in cardiomyocytes [1].

Tead proteins (Tead1-4) are ubiquitously expressed in all organs in a spatial and temporal manner [8]. Global deletion of Tead1, in mice, caused *in utero* lethality, at embryonic day 11.5, due to myocardial hypoplasia, but without overt disturbances in cardiac patterning, indicating its non-redundant role in early embryonic cardiomyocyte proliferation and cardiac development [9]; while deleting both Tead1 and Tead2 led to severe morphological defects at embryonic day 8.5 with a failure of heart tube formation [10]. However, whether Tead1 is required for normal cardiomyocyte proliferation at a later stage, such as in the perinatal period, is not known. We have recently generated mice carrying the Tead1 floxed allele to inducibly delete Tead1 and study its function later developmental and postnatal stages. We demonstrated that Tead1 had a critical function in adult cardiomyocytes and that its loss of function leads to rapid-onset severe dilated cardiomyopathy [11]. We now asked the question if Tead1 is required for cardiomyocyte proliferation in the perinatal period, when cardiomyocyte proliferation is very high, by deleting Tead1, perinatally, in cardiomyocytes using Myh6-Cre.

Our results demonstrated that cell-autonomous Tead1 function in cardiomyocytes was critical and its loss of function led to lethality by postnatal day 9, accompanied by significantly reduced cardiomyocyte proliferation, as well as systolic dysfunction and dilated cardiomyopathy. This was secondary to significant decrease in the levels of many cell cycle related proteins that promote cell cycle and proliferation. These results demonstrate that Tead1 is required and has a critical role in promoting cardiomyocyte proliferation in the perinatal period.

## Materials and methods

### Animal experiments

All animal procedures were performed at the Baylor College of Medicine, approved by the Institutional Animal Care and Use Committee (IACUC) of Baylor College of Medicine conforming to the Guide for the Care and Use of Laboratory Animals published by the U.S. National Institutes of Health. The conditional *Tead1*^F/F^ allele was constructed as previously described [11]. Cardiomyocyte-specific deletion of Tead1 (*Tead1*-cKO) was achieved by crossing *Tead1*^F/F^ mice with *Myh6-*Cre mice (011038, The Jackson Laboratory). Mice were maintained under standard 12-h light-dark cycles with ad lib access to food and water, unless specified otherwise. For tissue collection, mice were euthanized after isoflurane anesthesia, per approved protocols by the IACUC. To analyze the survival rate of *Tead1*-cKO pups, time to death (day)–when pups are found dead in the cage–was monitored and used as the endpoints. Animal health and behavior were monitored daily by trained researchers or veterinarians, no signs of severe suffering or distress were observed. Kaplan–Meier survival curves were generated from *n* of 15 for each group.

Under 1.0% isoflurane anesthesia, echocardiographic examination was conducted on the mouse pups using Vevo2011. End systolic and diastolic dimensions were obtained from M-mode ultrasound, ejection fraction and fractional shortening were calculated accordingly. Echocardiograms were performed blinded to genotype.

### Antibodies for immunoblotting and immunofluorescence

The antibodies used for immunoblotting are listed below: Tead1 [EPR3967(2)] (ab133533, Abcam; 1:2,000), Hsp90 (4874S, Cell Signaling; 1:2,000), Cell Cycle Regulation Antibody Sampler Kit (9932, Cell Signaling; 1:1,000 for all) & Sampler Kit II (9870, Cell Signaling; 1:1,000 for all). The antibodies for immunofluorescence are: cardiac Troponin T [1C11] (ab8295, Abcam; 1:500), Tead1 [EPR3967(2)] (ab133533, Abcam; 1:100), phospho-Histone H3 (Ser10) (9701, Cell Signaling; 1:100), Ki67 (ab16667, Abcam, 1:200).

### Histology

For Masson’s Trichrome staining, dissected hearts were immediately fixed in 10% formalin overnight at room temperature, followed by paraffin embedding. Coronal or cross sectioned tissues (5 μm) were deparaffinized in Histo-Clear (National Diagnostics), rehydrated and fixed in Bouin’s Solution at 56°C for 1 hr. Following washes in deionized water, sections were sequentially stained with Weigerts’ Iron Hematoxylin, Beibrich Scarlet-Acid Fuchsin solution, phosphotungstic/phosphomolybdic acid and Aniline blue (25088, Masson’s Trichrome stain kit from Polysciences). Sections were dehydrated via ethanol series, cleared in Xylene and mounted in resin-based medium. Images were analyzed using a Zeiss Axioplan-2 imaging system.

For wheat germ agglutinin (WGA) staining of tissue sections, following antigen retrieval in boiling 10mmol/L sodium citrate (pH 6.0) for 30min, sections were blocked with 10% goat serum, followed by incubation of the sections with Rhodamine-WGA (RL-1022, Vector Lab) at 5µg/ml and mounted with ProLong (ThermoFisher). Terminal deoxynucleotidyl transferase (TdT) dUTP Nick-End Labeling (TUNEL) assay was carried out following the manufacturer’s protocol using *In Situ* Cell Death Detection Kit (11684817910, Roche). For cytology staining, cells were washed with PBS and fixed in 10% formalin for 15 min at room temperature, incubated in 0.5% Triton X-100/PBS and blocked in 2% BSA/PBS. Cells were then incubated with primary antibodies as indicated, subsequently with Alexa Fluor conjugated anti-mouse IgG (Invitrogen, 1:200). Images were taken with a DeltaVision (Deconvolution) image restoration microscope or a Zeiss Axioplan-2 imaging system.

### Mouse cardiomyocytes culture

Mouse primary cardiomyocytes were isolated from the hearts of 0–3-day-old neonatal mice as described previously [12]. *Ex vivo Tead1* knockout in neonatal cardiomyocytes was achieved as previously described [11]. Mouse HL-1 cardiac muscle cell line (SCC065, Sigma-Aldrich) was maintained according to manufacturer’s protocol. HL-1 stable knockout cell lines were generated as previously described [13]. Briefly, Cas9 (blasticidin resistant) and Tead1-targeted sgRNA (sgRNA sequence: TAGCCAGATACATCAAACTC; puromycin resistant) lentiviruses were transduced to generate the Tead1 knockout cells, followed by blasticidin 1μg/ml and puromycin 1μg/ml selection for 3 weeks. Similarly, Cas9-control cells were generated by transduction of Cas9-lentivirus only and followed by blasticidin selection. Knockout efficiency was validated by further experimentation. LentiCas9-Blast was a gift from Feng Zhang (Addgene plasmid # 52962).

### RNA extraction and real-time PCR

Total RNAs were extracted and cleaned up using RNeasy Mini Kit (QIAGEN) according to manufacturer’s protocol. Complementary DNA were synthesized using q-Script cDNA Supermix kit (Quanta Biosci.). Realtime PCR was performed using Perfecta SYBR Green Supermix (Quanta Biosci.) in a Roche 480 Light Cycler machine. Relative mRNA expression levels were determined by normalization of target genes to 36B4 as internal control. Primer sequences are available upon request.

### Western blot analysis

Tissue protein samples were prepared in ice-cold lysis buffer (100 mmol/L TrisHCl pH7.4, 10 mmol/L NaCl, 1 mmol/L EDTA, 1 mmol/L EGTA, 10% glycerol, 0.5% deoxycholate, 1% Triton X-100, 0.1% SDS, 20mmol/L Na_4_P_2_O_7_, 2 mmol/L Na_3_VO_4_, 1 mmol/L NaF, 1 mmol/L PMSF) supplemented with 1X protease inhibitor and phosphatase inhibitor (Roche). Equal amount of protein samples was loaded and fractionated by SDS-PAGE, then transferred to 0.45 μm pore-size nitrocellulose membranes (Millipore). Membranes were blocked in 5% non-fat milk-TBS (w/v), then incubated with indicated primary antibodies and HRP-conjugated secondary antibodies, enhanced chemiluminescence substrate (Thermo Sci.) was applied for detection. Band densities were quantified by ImageJ software.

### Statistical analysis

The data are presented as means ± s.e.m. Significance tests were determined by the two-tailed, unpaired Student’s *t* test, or two-way ANOVA followed by Sidak’s multiple comparisons test. p< 0.05 was considered statistically significant. GraphPad Prism 7.04 software was used for statistical analyses.

## Results

### Cardiomyocyte-specific deletion of Tead1 leads to early neonatal death

**Fig 1:**
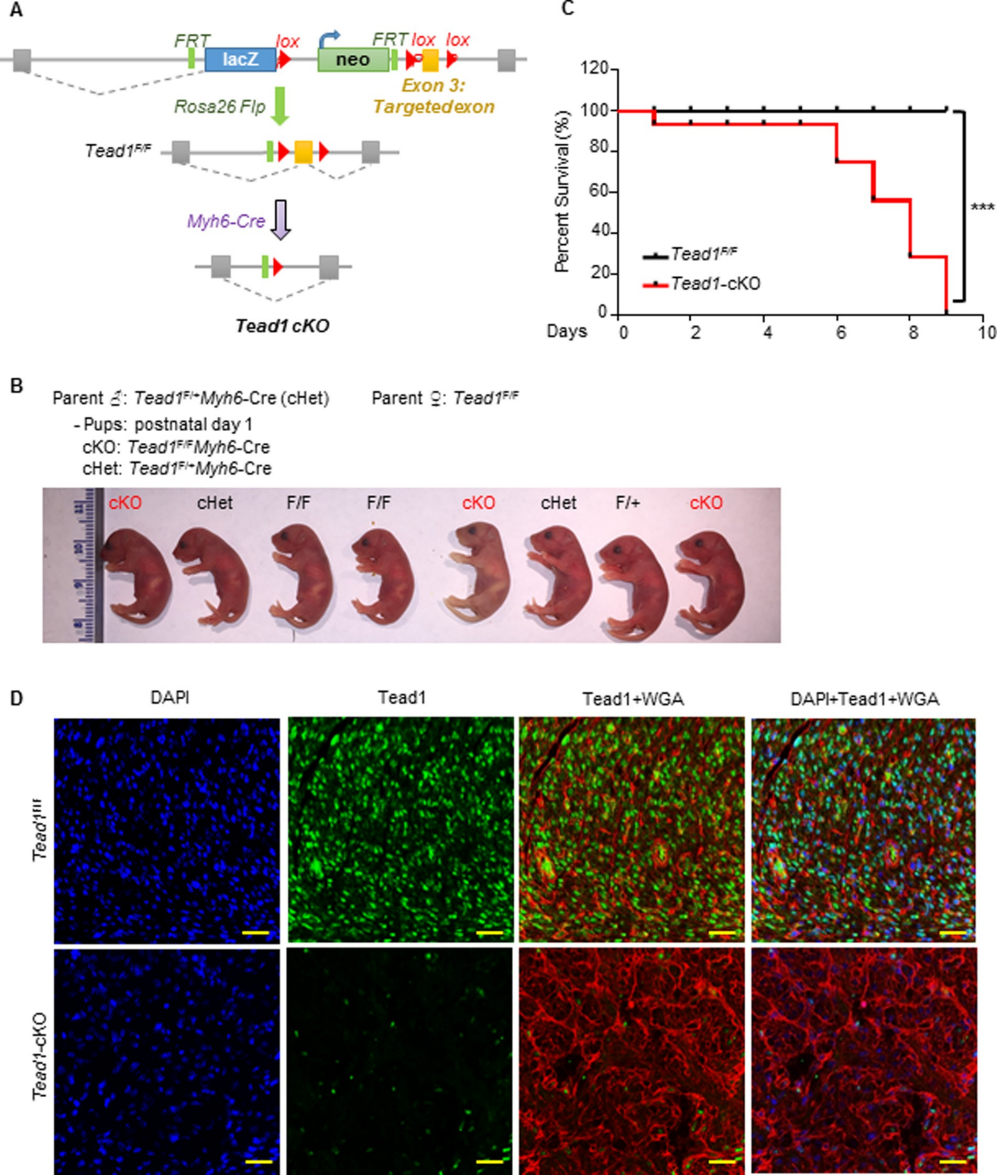
Cardiomyocyte-specific deletion of *Tead1* leads to early neonatal death by day 9 after birth. (**A**) Generation of *Tead1* cardiomyocyte-specific knockout mice (*Tead1*-cKO) using knockout-first strategy: promoter-driven selection cassette. (**B**) Representative photograph of a *Tead1*-cKO breeding litter, demonstrating *Tead1*-cKO were born in Mendelian manner and not overtly distinguishable from littermates. (**C**) Kaplan-Meier survival curves of *Tead1*^*F/F*^ littermates and *Tead1*-cKO mice. n = 15 mice per group. *** *p*<0.001 by log-rank (Mantel-Cox) test (**D**) Immunostaining in day 1 heart section verifying Tead1 deletion. Scale bar, 50μm.

Global deletion of Tead1 leads to fetal demise at embryonic day e11.5 [9], precluding the investigation of its function in later cardiac developmental stages. We have previously generated mice with conditional Tead1 floxed alleles (Tead1^F/F^) and have demonstrated that Tead1 is essential in the regulation of adult cardiomyocyte function, using an inducible knockout mouse model [11], wherein Tead1 was deleted only in adult cardiomyocytes in 5 week old mice. We also found that Tead1 expression peaked during late embryonic and early postnatal stages, which correspond with periods of high proliferation in cardiomyocytes [11]. Therefore, to determine if Tead1 plays a non-redundant role in the perinatal heart, when proliferation of cardiomyocytes is high, we generated mice with a cardiomyocyte-specific deletion of Tead1, using myosin heavy chain 6 (*Myh6*)-Cre deletor mice, when cardiac patterning and chamber formation are completed [14] (hereafter referred as *Tead1*-cKO; Fig 1A). *Tead1*-cKO pups were born at Mendelian frequency, overtly indistinguishable at birth from littermates (Fig 1B). However, after postnatal day 5, there was increased mortality in the Tead1-cKO pups, and none survived beyond postnatal day 9, as shown in the survival curve in Fig 1C (*p*<0.001). Thus, deletion of Tead1 in perinatal cardiomyocytes led to 100% mortality with early neonatal death.

Immunostaining of the heart ventricular sections from 1 day old pups showed nuclear expression of Tead1 co-localizing with Troponin T staining in control mouse cardiomyocytes and an efficient Tead1 deletion in Tead1-cKO cardiomyocytes (Figure A in S1 File). WGA and DAPI co-staining suggested disorganized myofibers in Tead1-cKO compared to flox control hearts (Fig 1D). These results demonstrated Tead1 function in the cardiomyocytes is non-redundant in the maintenance of postnatal viability.

### Tead1 loss-of-function results in neonatal dilated cardiomyopathy

**Fig 2:**
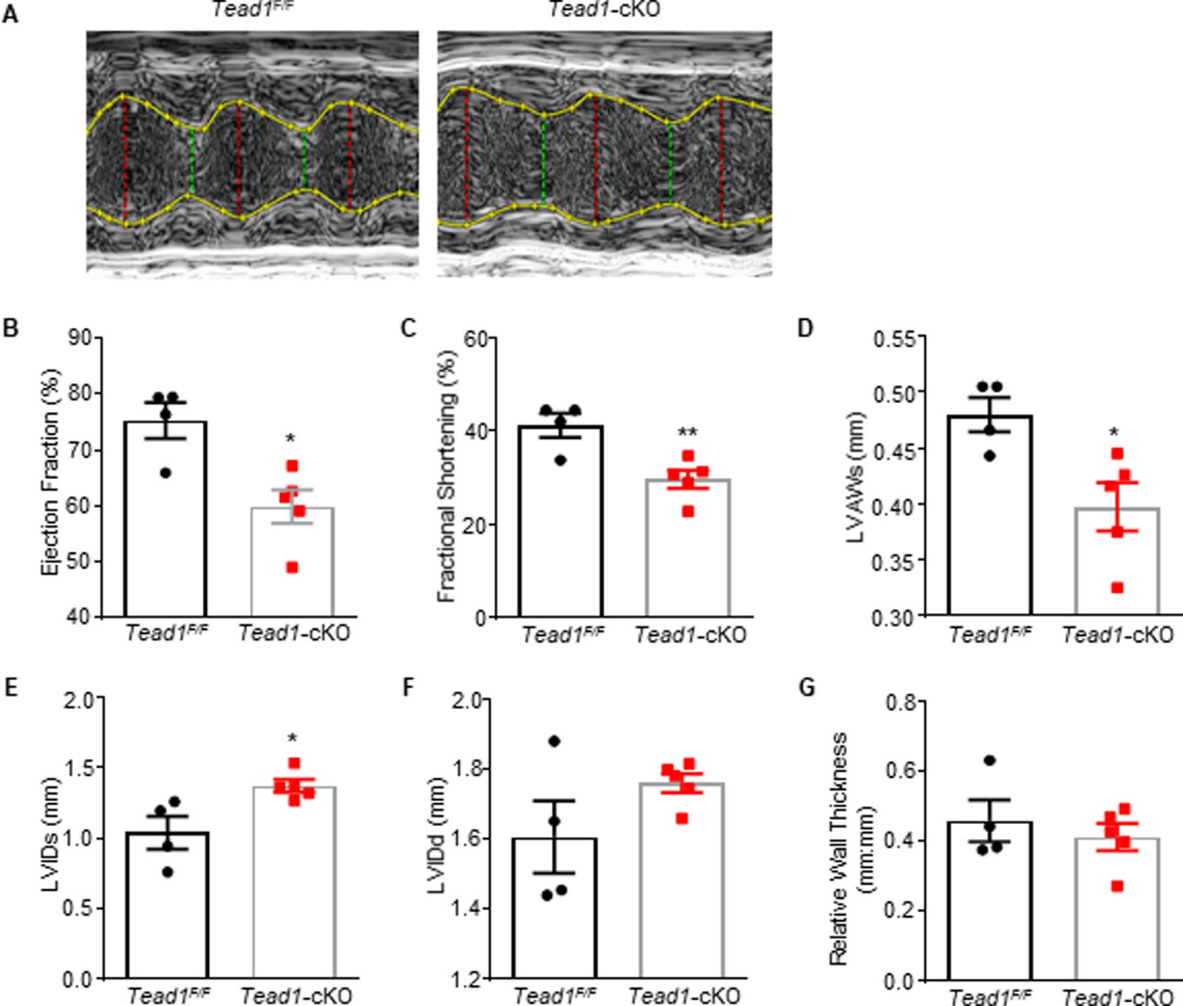
Tead1 is required for postnatal cardiac function. Echocardiography on postnatal day 1 neonatal pups. (**A**) Representative image of the echocardiography. (**B**) ejection fraction, (**C**) fractional shortening (FS), (**D**) left ventricular anterior wall at systole (LVAWs), (**E-F**) left ventricle internal diameter at systole and diastole (LVIDs and LVIDd), and (**G**) relative wall thickness (RWT, 2xLVPWd/LVIDd) of the *Tead*^*F/F*^ and *Tead1*-cKO pups (n = 4–5). **p < 0.01, *p < 0.05; Student’s *t*-test.

To evaluate the systolic function of Tead1 deficient hearts, M-mode echocardiography on left ventricles was performed in 1-day-old pups (Fig 2A). Ejection fraction and fractional shortening were significantly decreased by 20.4% (*p* = 0.01) and 28.0% (*p* <0.01), respectively, in Tead1-cKO pups compared with flox littermates (Fig 2B and 2C). The left-ventricular anterior wall thickness (LVAWs; 0.39 ± 0.02 mm compared with 0.48 ± 0.02 mm, *p* = 0.02) was lower (Fig 2D), while internal diameter (LVIDs; 1.37 ± 0.04 mm compared with 1.04 ± 0.12 mm, *p* = 0.0220) at systole, was significantly higher, as was the left-ventricular internal diameter at diastole (LVIDd), which showed a trend towards being increased (1.76 ± 0.03 mm compared with 1.61 ± 0.10 mm, *p* = 0.15) (Fig 2E and 2F). The calculated relative wall thickness (2 X posterior wall thickness / left ventricular diastolic diameter) was unchanged (0.41 ± 0.04 mm:mm compared with 0.46 ± 0.06 mm:mm, *p* = 0.52) (Fig 2G). In all, the echocardiographic analyses indicated that *Tead1*-cKO pups showed significant left ventricular systolic dysfunction and displayed features of chamber enlargement, consistent with dilated cardiomyopathy (DCM).

**Fig 3:**
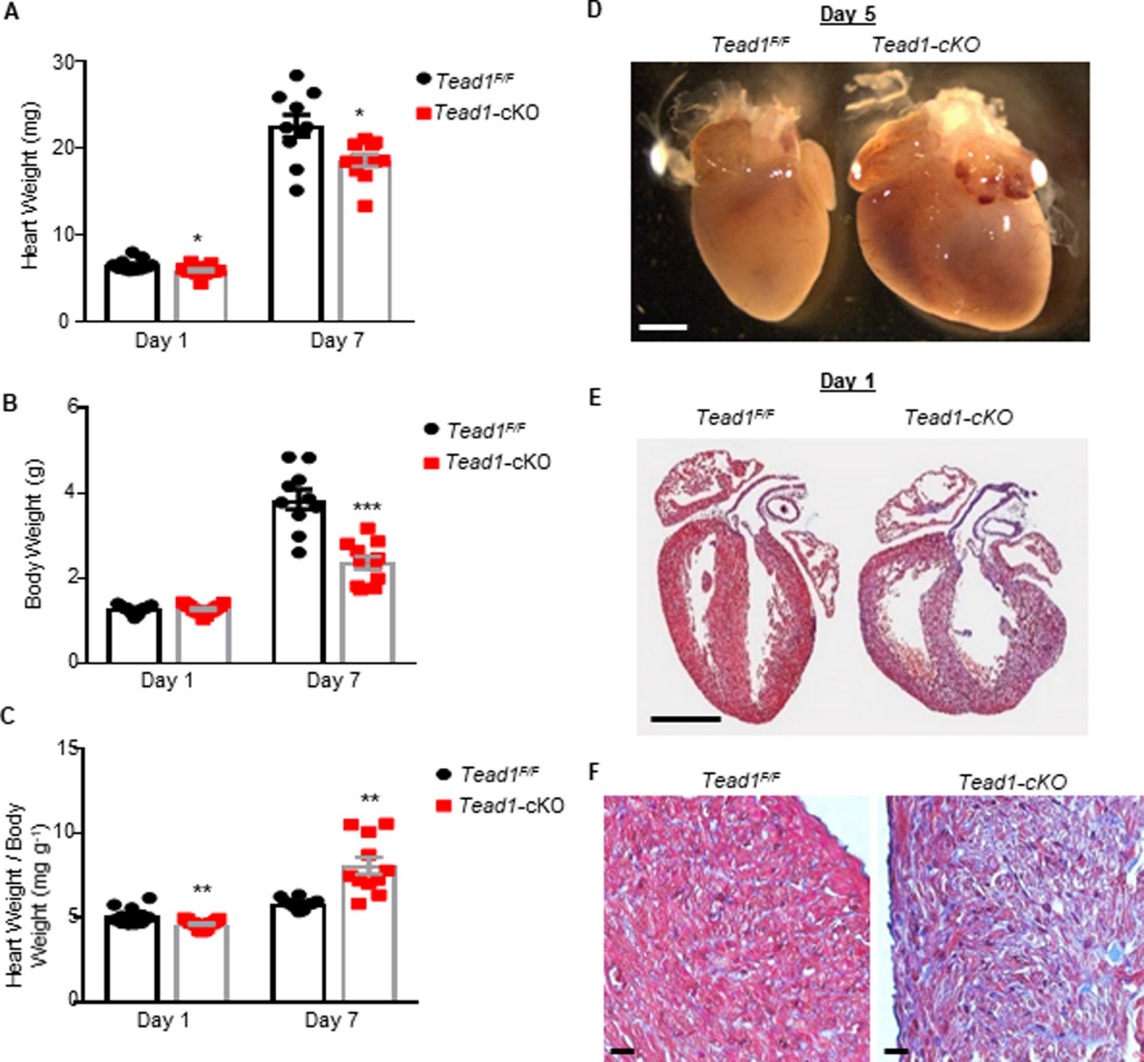
Tead1 loss-of-function results in heart failure associated with myocardium hypoplasia. (**A**) Heart weight (**B**) Body weight and (**C**) heart weight to body weight ratios of Tead1-cKO mice at postnatal days 1 and 7 (n = 10–13). ***p < 0.001, **p < 0.01; Student’s *t*-test. (**D**) *Tead*^*F/F*^ and *Tead1*-cKO postnatal days 5 hearts. Scale bar, 1mm. (**E-F**) Representative coronal section Trichrome staining of day-1 *Tead*^*F/F*^ and *Tead1*-cKO mice hearts. Scale bar, 1mm (**E**), 20μm (**F**).

As shown in Fig 3A–3C, heart weight of 1-day-old Tead1-cKO pups was significantly lower compared to flox littermates (5.92 ± 0.169 g compared with 6.54 ± 0.20 g, *p* = 0.03), though the body weight was the same (1.28 ± 0.03 g compared with 1.28 ± 0.03 g, *p* = 0.95). However, the heart weight normalized to body weight also being significantly lower in Tead1-cKO (5.120 ± 0.153 mg/g compared with 4.62 ± 0.07 mg/g, *p* = 0.005). As the pups grew older, heart weight and body weight of the floxed controls rose significantly, while this rise was significantly less in the Tead1-cKO pups, such that at postnatal day 7, the Tead1-cKO pups heart (18.546 ± 0.67 g compared with 22.480 ± 1.28 g, *p* = 0.01) and body weight (2.37 ± 0.15 g compared with 3.85 ± 0.23 g, *p* < 0.001) was significantly less than their floxed control pups. Since the rise in body weight was far lower in the Tead1-cKO pups their normalized heart weight to body weight became significantly higher than the control flox littermates (8.075 ± 0.501 mg/g compared with 5.848 ± 0.100 mg/g, *p* = 0.0005), a finding consistent with their ongoing heart failure phenotype.

A significant enlargement of the *Tead1*-cKO hearts was observed by postnatal day 5, as shown in Fig 3D. Trichrome staining of the heart on day 1 old *Tead1*-cKO pups did not reveal any structural abnormalities in chamber development consistent with echocardiographic studies (Fig 3E). Along with chamber enlargement, a significant increase in intercellular fibrotic reaction was evident on Trichrome staining in *Tead1*-cKO pups, as early as postnatal day 1 (Fig 3F).

### Molecular markers of fetal program following Tead1 ablation in cardiomyocytes

**Fig 4:**
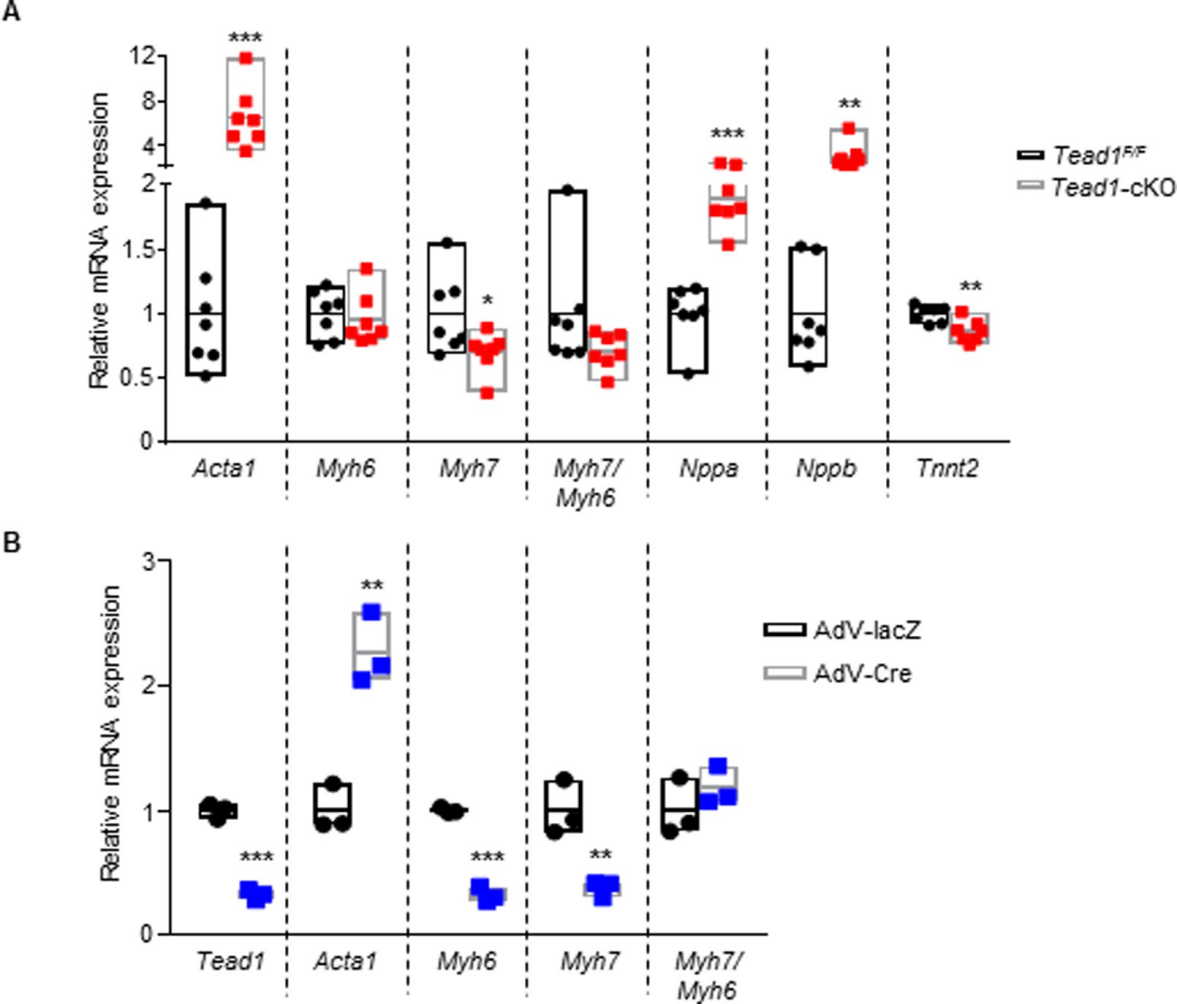
Tead1 deletion in perinatal cardiomyocytes leads to elevated markers of heart failure. Data are presented as mean ± s.e.m. ***p < 0.001, **p < 0.01, *p < 0.05; data were analyzed by Student’s *t*-test. Comparisons between (**A**) *Tead1*-cKO pups and *Tead*^*F/F*^ pups (n = 7), (**B**) *Tead*^*F/F*^ neonatal cardiomyocytes transduced with adeno-virus Cre (Tead1 deletion) or lacZ (control).

To characterize the mechanisms underlying the regulatory role of Tead1 in cardiomyocytes in the perinatal period, we assessed changes in gene expression in Tead1 knockout hearts. RNA isolated from the ventricles from 1-day-old Tead1-cKO and control flox littermate pups was assayed for molecular markers associated with heart failure and the cardiac fetal program (Fig 4A). Many of the markers often associated with heart failure were differentially changed with Tead1 deletion. Changes in α-skeletal actin (*Acta1–*6 fold increase), atrial natriuretic peptide (*Nppa*– 1.9 fold increase), brain natriuretic peptide (*Nppb*– 2.6 fold increase), cardiac troponinT (cTnT– 14% decrease), consistent with expected molecular signature of heart failure. Heart failure-associated fetal gene program is also characterized by upregulation of β-MyHC (*Myh7*), the dominant isoform of myosin heavy chain in the fetal cardiomyocytes, and a down regulation of α-MyHC (*Myh6*), the dominant isoform in the adult cardiomyocytes [15]. However, in the Tead1-cKO pups, despite a DCM phenotype, *Myh7* was not upregulated, and was in-fact downregulated significantly by 30%, compared to control flox littermates. To confirm that these changes were primarily due to the cell-autonomous loss-of-function of Tead1, and not secondary (non-cell autonomous) changes from heart failure, we used our, previously described, *ex vivo* knockout model [11], wherein we compared gene expression changes with Tead1 deletion ex vivo, in Tead1^F/F^ neonatal cardiomyocytes treated with Ad-Cre, as compared to that with control Ad-lacZ treatment. Ex vivo Tead1 deletion in these neonatal cardiomyocytes mirrored the in vivo Tead1 deletion findings of up-regulation of α-skeletal actin (*Acta1*) and a down-regulation of both α-, β-MyHC (*Myh6*, *Myh7*) (Fig 4B), suggesting Tead1 regulation of these fetal program genes [15] in cardiomyocyte is unique and cell-autonomous and that tead1 is required for β-MyHC upregulation in heart failure.

### Tead1 is required for cardiomyocyte proliferation

**Fig 5:**
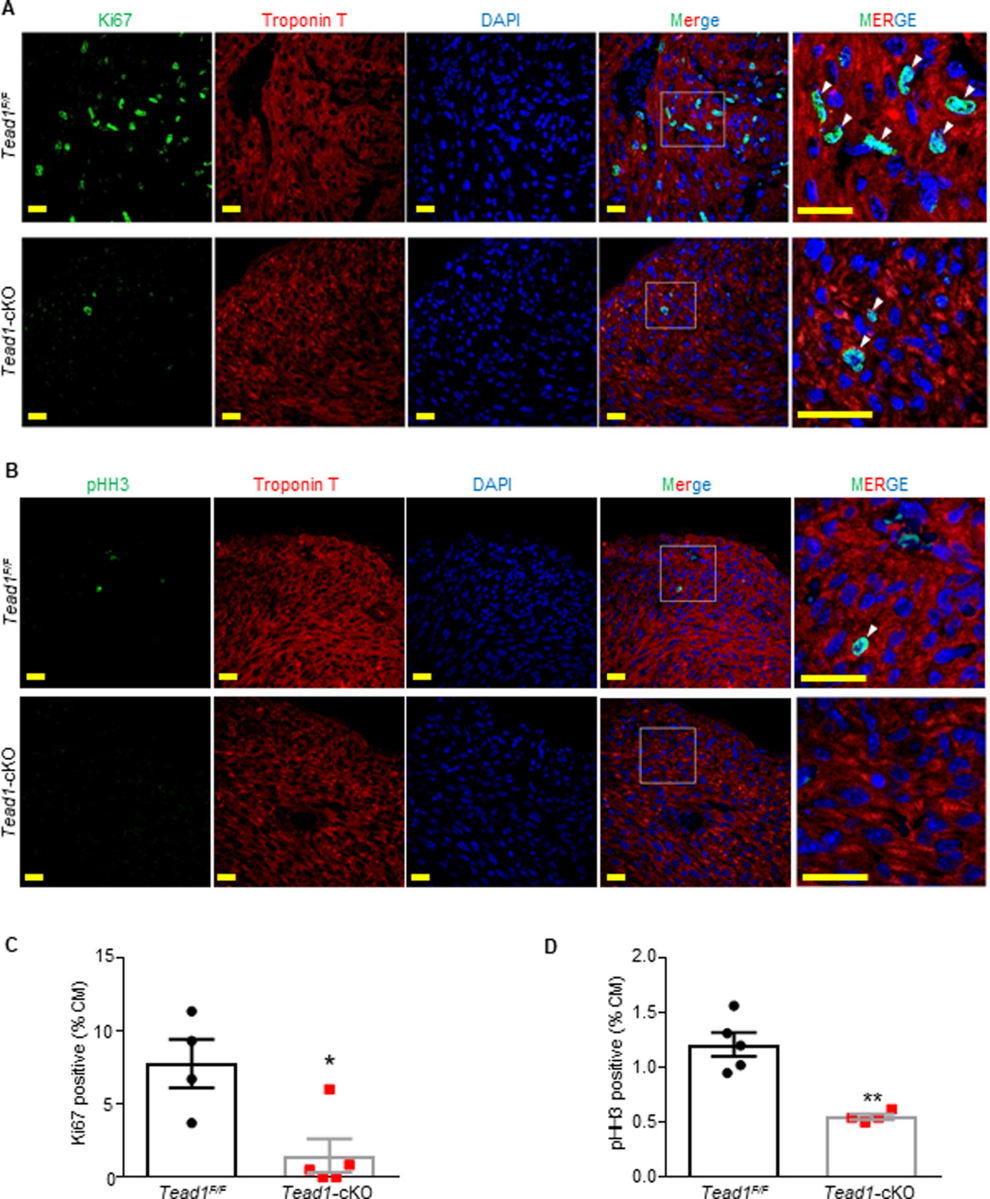
Tead1 is required for cardiomyocyte proliferation. (**A, B**) Cardiomyocyte proliferation (Ki67) and mitosis (phospho-Histone 3^Ser10^ positive immunostaining) in postnatal day 1 *Tead*^*F/F*^ and *Tead1*-cKO hearts. (**C, D**) Quantitative analysis represents counting of multiple sections from three independent samples per group (approximately 1.2–1.5 X 10^5^ cardiomyocytes were counted). **p < 0.01; Student’s *t*-test. Scale bar, 20 μm.

We next sought to test if cardiomyocyte proliferation was impaired in *Tead1*-cKO pups. Detection for cell proliferation in heart sections from 1-day-old pups, by immunostaining for Ki67 (marker for G1/S/G2/M phases of the cell cycle) (Fig 5A), indicated that there were significantly less (82% reduction) number of Tead1-deficient cardiomyocytes entering cell cycle (1.4 ± 1.14% Ki67 pos CMs in Tead1-cKO compared with 7.7 ± 1.65% in floxed controls, *p* = 0.03) (Fig 5C). Similarly, decreased (45% reduction) number of phospho-Histone H3 at serine 10 (pHH3 –marker for M phase) positive cardiomyocytes in heart sections from 1-day-old pups (0.5 ± 0.03% pHH3 positive CMs in Tead1-cKO compared with 1.2 ± 0.11% in floxed controls, *p* = 0.0012) indicated that there was a decrease in number of cells entering mitosis in the Tead1-cKO hearts (Fig 5B and 5D). We also assessed for apoptosis by the terminal deoxynucleotidyl transferase (TdT) dUTP nick-end labeling (TUNEL) assay and we did not detect a difference in apoptosis in either cKO compared to floxed control hearts (Figure B in S1 File). These results demonstrated that Tead1 is required for cardiomyocytes to enter cell cycle proliferation during the perinatal period.

To confirm that Tead1 regulated cell proliferation in a cell-autonomous manner, we established stable mouse HL-1 cardiac cell line with Tead1 deletion (Figure C (i) in S1 File) using CRISPR/Cas9 system and characterized cell proliferation. Tead1-KO and control cells were synchronized at G0 phase by serum depletion and cell numbers were determined every 48 hours. Growth curves demonstrated a significant decrease in Tead1-KO cells compared to control cells, by 37% on day 4 and day 6 (Figure C (ii) in S1 File). This result confirmed that Tead1-regulation of cell cycle is cell-autonomous.

### Loss of Tead1 leads to decreased levels of critical cell cycle proteins

**Fig 6:**
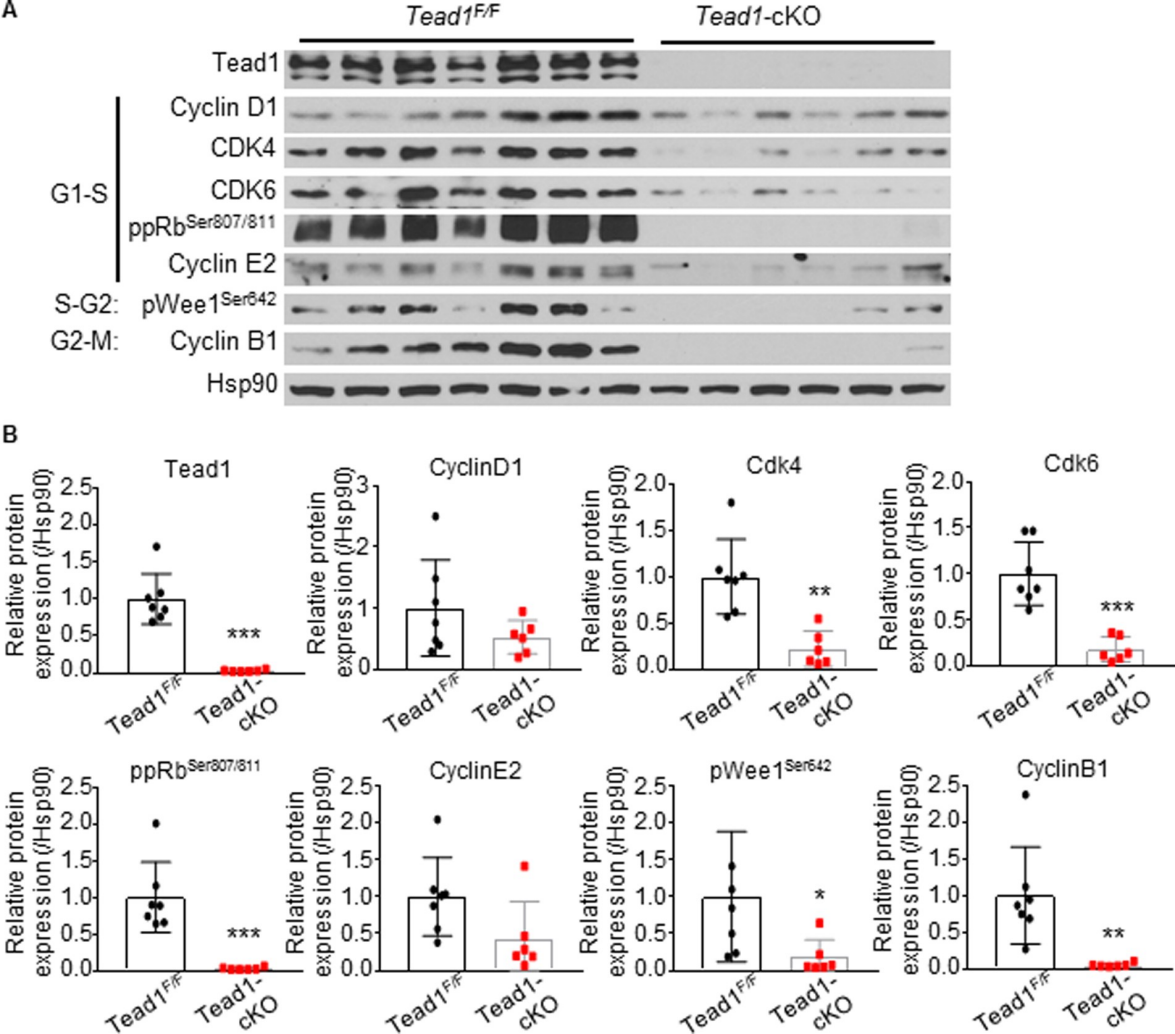
Loss of Tead1 leads to decreased expression of cell cycle proteins. **(A)** Western blot analysis of cell cycle related proteins in postnatal day 7 *Tead*^*F/F*^ (n = 7) and *Tead1*-cKO (n = 6) hearts. Loading control, Hsp90. **(B)** Densitometry analyses of relative protein expression, normalized by Hsp90. Data are presented as mean ± s.e.m. ***p < 0.001, **p < 0.01, *p < 0.05; data were analyzed by Student’s *t*-test.

The expression of essential cell cycle proteins was assessed in *Tead1*-deleted myocardium as compared to control flox littermates, by Western blotting. There was a significant decrease in the levels of G1/S phase regulating proteins, Cdk4, Cdk6, hyperphospho-Rb^Ser807/811^ levels, which suggested that Tead1 is required for cardiomyocytes to progress through G1-S phase. Similarly, the levels of phospho-Wee1^Ser642^ and cyclin B1, which regulate cell cycle progression from S to G2 and G2/M phases, were decreased in Tead1-cKO hearts (Fig 6A and 6B).

Taken together, these data demonstrate that Tead1 is critical for the proliferation of CM perinatally via regulation of the levels of many critical cell cycle markers in all phases of cell cycle and its perinatal loss leads to heart failure and DCM.

## Discussion

In the present study, we examined the role of Tead1 in proliferating cardiomyocytes by generating perinatal cardiomyocyte-specific *Tead1* knockout mice using Myh6-Cre- mediated deletion of Tead1 floxed alleles. None of the *Tead1*-cKO mice survived beyond postnatal day 9 and died of severe heart failure. Decreased expression of cell cycle promoting genes accompanied by decreased cardiomyocyte proliferation was observed, demonstrating the non-redundant role of Tead1 in proliferating cardiomyocytes in the perinatal period. In addition, absence of upregulation of βMHC (Myh7), a fetal gene program marker of heart failure, in the failing hearts of the *Tead1*-cKO mice demonstrates requirement of Tead1 for heart failure-associated fetal program increase in βMHC.

Canonical Hippo-Tead signaling consists of a kinase cascade, modulated by both extra- and intra-cellular cues to regulate multiple cellular functions, including, cell proliferation. Previous studies on Hippo signaling in the heart have demonstrated that upstream components of the mammalian Hippo pathway, including Mst1/2, Sav, Lats1/2 all negatively regulate cardiomyocyte proliferation [3, 16–19], while Yap was shown to be a positive regulator of cardiomyocyte proliferation [4]. While Tead1 has been shown to indirectly mediate the effects of upstream Hippo components, there has not been any definitive study to demonstrate the role of Tead1 in cardiomyocyte proliferation in the perinatal period.

Heart specific inactivation of Yap1, using early cardiac developmental marker-driven Cre lines–such as *Nkx2*.*5*, *Myh7* and *Tnnt2* –are embryonic lethal due to decreased CM proliferation [1, 4]. While using the *Myh6*-Cre, expressed at embryonic day 10.5 and upregulated during fetal and postnatal cardiac development, to inactivate Yap, death occurred only when the mice were 11 to 20-week-old, with dilated cardiomyopathy. In the same study, Yap was also shown to be critical for neonatal cardiac regeneration [5]. While, Taz knockout mice, using *Myh6*-Cre, were viable without evident cardiac impairment, deletion of both cardiac Yap and Taz led to death by post-natal day 1, significantly earlier than when either of the two genes was deleted alone, indicating redundancy between the functions of Yap and Taz, as well as possible gene dosage dependent effects [5]. Since, both Yap and Taz act as co-activators of Tead1, early neonatal death due to Yap/Taz combined deletion is consistent with our observation of early neonatal death secondary to heart failure with decreased cardiomyocyte proliferation after Tead1 deletion in cardiomyocytes, using the same *Myh6*-Cre line. It is possible that the slightly earlier death seen in cardiomyocyte specific Yap/Taz double knockout mice could be due to the loss of function of critical Tead1-dependant [1], and Tead1-independent [7, 20] Yap/Taz regulated pathways.

Though it has been suggested that Tead1 mediates the effects of upstream Hippo components in the heart, this has largely been based only on supportive evidence. The only direct evidence for a role of Tead1 in CM proliferation was seen with the global Tead1 KO, which die embryonically, at e11.5 [9], from cardiac hypoplasia due to a decrease in CM proliferation. While this demonstrated the role of Tead1 in embryonic CM proliferation, there has not been any study to demonstrate a role for Tead1 in subsequent CM proliferation in the perinatal period. In this study we used the Myh6-Cre deletor line to address this. The expression of Myh6-Cre in the heart has been reported to be as early as e8.5 [21], predominantly in atria, with ventricular expression being lower until just before birth, when it rises significantly. Hence Myh6 promoter-driven Cre mediated recombination in ventricular cardiomyocytes could have incomplete and variable efficacy prior to birth. At e14.5, the expression of Tead1 in cardiomyocytes was variable in our *Tead1*-cKO mice with some CMs with Tead1 deletion and others with persistent presence (data not shown), unlike that seen at P1 pups (Fig 1 and Figure A in S1 File), wherein most CMs had deletion of Tead1. Hence, while we can conclude that Tead1 is required for CM proliferation in the perinatal period, the Myh6-Cre deletor model cannot address whether Tead1 is required in the embryonic periods.

Our data demonstrated significantly decreased levels of cardiomyocytes that were entering cell cycle, in vivo, along with a significant decrease in many proteins that promote progression through the various stages of the cell cycle, in Tead1 ablated myocardium, consistent with that observed in vitro, in Tead1-deficient HL1 cells. Based on these findings, we infer that Tead1 is required for cardiomyocyte cell cycle progression and that lack of cell-autonomous Tead1 function leads to cell cycle withdrawal. These data are also consistent with previous studies in other cell types. Tead1 germline mutant embryos presented decreased percentage of myocardial cells in S phase [20] as was also seen in breast cancer cells, wherein knockdown of all Tead family members led to similar cell cycle changes [22]. Interestingly, Tead1 overexpression in cardiomyocytes, in vivo, was not sufficient to induce significant hyperplasia of the myocardium [23]. The lack of hyperplasia with Tead1 overexpression, in vivo, taken together with our current findings indicate that Tead1 is required, but not sufficient for cardiomyocyte proliferation.

We had previously demonstrated that in adult cardiomyocytes Tead1 is a direct transcriptional regulator of Ppp1r1a (Inhibitor-1) and that loss-of-function of Tead1 leads to increased PP1 activity, decreased (PLN) phospholamban phosphorylation and consequent decrease in Serca2a activity, along with heart failure [11]. In the current study, we observed a similar decrease in Inhibitor-1 and decreased phosphorylation of phospholamban (data not shown), suggesting that this is a conserved mechanism even in neonatal CMs and may contribute to the observed early lethality observed in the Tead1 cKO mice.

Heart adaptation to pathophysiologic insults, such as myocardium ischemia, pressure/volume overload, often includes an induction of a ‘fetal gene program’ [24]. In rodent myocardium the predominant isoform of myosin heavy chain (MHC) is the β-MHC and post-natally this transitions to the α isoform so that in the adult rodent myocardium α-MHC is the predominant isoform. An isoform switch from α-MHC to β-MHC, due to an increased expression of β-MHC with a concomitant repression of α-MHC, is a prominent feature in the stressed rodent myocardium. However, in the Tead1-depleted cardiomyocytes, ex vivo (ex vivo Tead1 deletion in neonatal cardiomyocytes) and in vivo (Tead1-cKO) models, both α-MHC and β-MHC are significantly repressed despite the evident heart failure phenotype in vivo. This is evidence that Tead1 is a transcriptional regulator of both α- and β-MHC [23, 25] and this demonstrates that cell-autonomous function of Tead1 is required for the ‘normal’ induction of the fetal gene program associated upregulation of β-MHC in the stressed rodent myocardium. In stressed myocardium, there is an induction of α-skeletal actin (*Acta1*), as was seen in Tead1 deletion models, both in vivo and ex vivo, indicating that this regulation of Acta1 by Tead1 is cell-autonomous and repressive. Repression of certain genes by Tead1 has been previously reported, especially in concert with repressors, such as Vgll4 [26] in the heart, and other cell types [27]. Tead1 has been reported to upregulate Acta1 during skeletal muscle differentiation [28] but a repressive regulation in cardiomyocyte has not been previously noted and will need to be addressed in future work.

In conclusion, our study demonstrates the critical non-redundant role of Tead1 in proliferating cardiomyocytes, in the perinatal period. Understanding the regulatory mechanisms underlying Tead1 function in cardiomyocyte proliferation could allow identification of new targets for promoting cardiomyocyte proliferation for regenerative therapies for heart diseases in the future.

## Supporting information

S1 File Supporting figures and legends. **Figure A. Cardiomyocyte Tead1 knockout validation on heart sections from 1.5-day-old pups.** Representative immunostaining showing Tead1 (green), cardiac Troponin T (red), DAPI (blue). Scale bar, 50 μm.
**Figure B. Tead1 ablation did not impact apoptosis.** (A) Representative figures of the TUNEL staining in control and Tead1-cKO heart sections. Scale bar, 100 μm. (B) Quantitative analysis represents counting from three independent samples per group (approximately 0.7–1.7 X 105cells were counted). Data were analyzed by Mann-Whitney t-test.
**Figure C. Tead1 regulates cardiomyocyte growth and cell cycle in a cell-autonomous manner.** (i) Western blot validation for Tead1 knockout in stable HL-1 cells. (ii) Cell counts in HL1-Cas9 and HL1-Tead1 knockout (HL1-Tead1KO) cells. Equal number of cells were plated and synchronized by overnight serum starvation, and cell numbers were counted at the indicated time points after reintroduction of complete growth medium (n = 4). ** p&lt; 0.01; *** p&lt; 0.001; data were analyzed by two-way ANOVA followed by Sidak’smultiple comparisons test.
(PDF)
