# Supplementary material for: Tead1 is required for perinatal cardiomyocyte proliferation
Source: PLoS One. 2019 Feb 27;14(2):e0212017. doi: 10.1371/journal.pone.0212017 (PMC6392249; doi:10.1371/journal.pone.0212017)
Supplement: S1 File — Figure A. Cardiomyocyte Tead1 knockout validation on heart sections from 1.5-day-old pups. Representative immunostaining showing Tead1 (green), cardiac Troponin T (red), DAPI (blue). Scale bar, 50 μm. Figure B. Tead1 ablation did not impact apoptosis. (A) Representative figures of the TUNEL staining in control and Tead1-cKO heart sections. Scale bar, 100 μm. (B) Quantitative analysis represents counting from three independent samples per group (approximately 0.7–1.7 X 105cells were counted). Data were analyzed by Mann-Whitney t-test. Figure C. Tead1 regulates cardiomyocyte growth and cell cycle in a cell-autonomous manner. (i) Western blot validation for Tead1 knockout in stable HL-1 cells. (ii) Cell counts in HL1-Cas9 and HL1-Tead1 knockout (HL1-Tead1KO) cells. Equal number of cells were plated and synchronized by overnight serum starvation, and cell numbers were counted at the indicated time points after reintroduction of complete growth medium (n = 4). ** p< 0.01; *** p< 0.001; data were analyzed by two-way ANOVA followed by Sidak’smultiple comparisons test. (PDF) [file pone.0212017.s001.pdf]

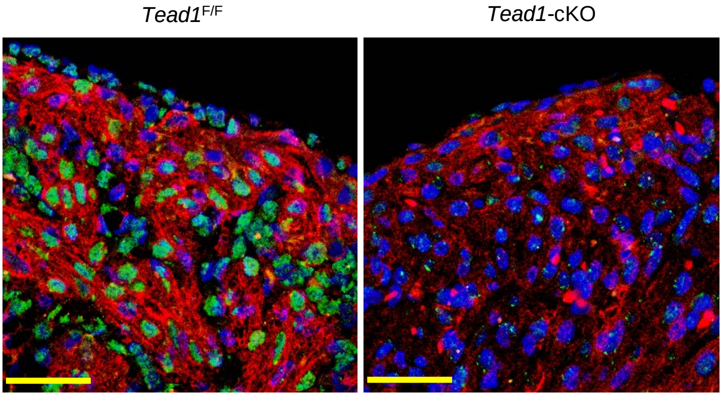

Figure A. Cardiomyocyte Tead1 knockout validation on heart sections from 1.5-day-old pups. Representative immunostaining showing Tead1 (green), cardiac Troponin T (red), DAPI (blue). Scale bar, 50 µm.

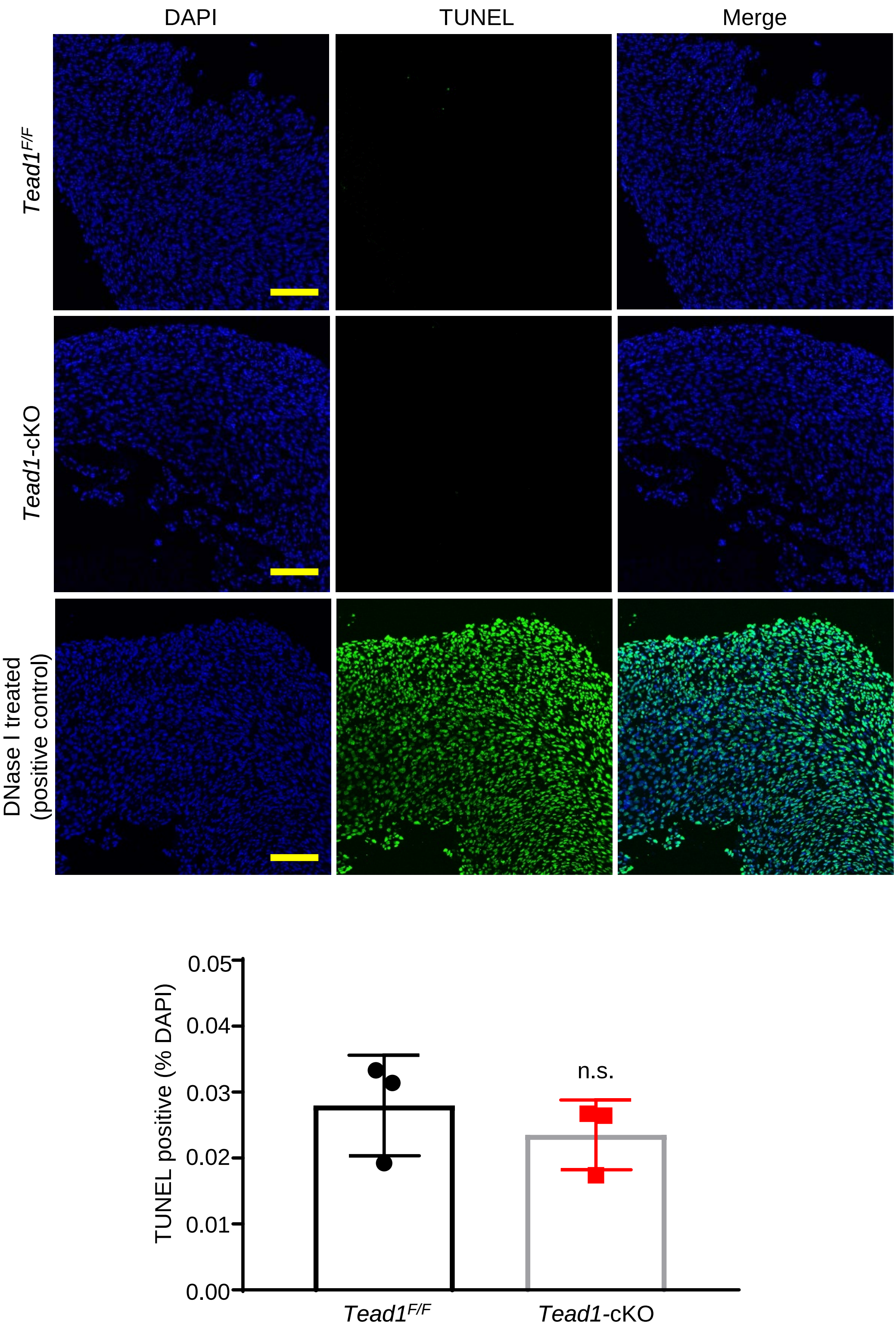

Figure B. Tead1 ablation did not impact apoptosis. (A) Representative figures of the TUNEL staining in control and Tead1-cKO heart sections. Scale bar, 100 µm. (B) Quantitative analysis represents counting from three independent samples per group (approximately 0.7-1.7 X 10^5 cells were counted). Data were analyzed by Mann-Whitney *t*-test.

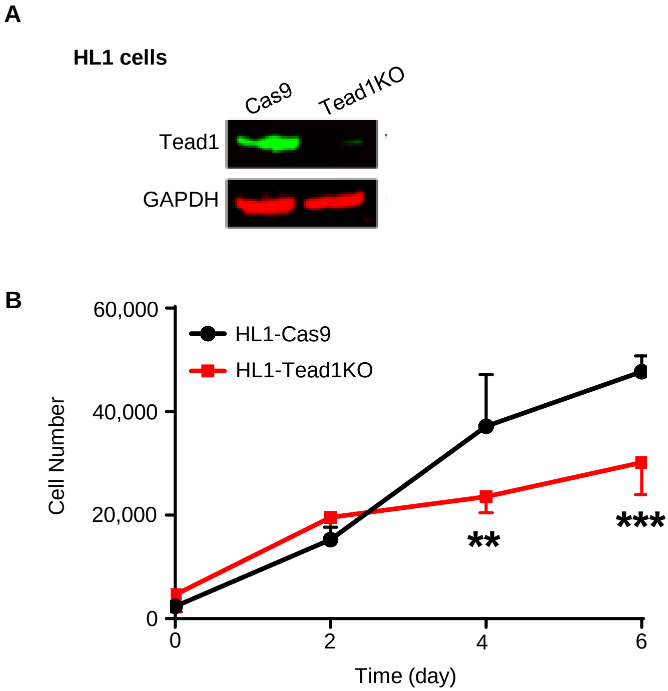

Figure C. Tead1 regulates cardiomyocyte growth and cell cycle in a cell-autonomous manner. (**i**) Western blot validation for Tead1 knockout in stable HL-1 cells. (**ii**) Cell counts in HL1-Cas9 and HL1-Tead1 knockout (HL1-Tead1KO) cells. Equal number of cells were plated and synchronized by overnight serum starvation, and cell numbers were counted at the indicated time points after reintroduction of complete growth medium (n=4). ** $p < 0.01$; *** $p < 0.001$; data were analyzed by two-way ANOVA followed by Sidak's multiple comparisons test.
